# Fabrication and Characterization of 3D-Printed Highly-Porous 3D LiFePO_4_ Electrodes by Low Temperature Direct Writing Process

**DOI:** 10.3390/ma10080934

**Published:** 2017-08-10

**Authors:** Changyong Liu, Xingxing Cheng, Bohan Li, Zhangwei Chen, Shengli Mi, Changshi Lao

**Affiliations:** 1 Additive Manufacturing Research Institute, College of Mechatronics and Control Engineering, Shenzhen University, Shenzhen 518060, China; liuchangyong@aliyun.com (C.Li.); gxchaun@gmail.com (X.C.); revendway@hotmail.com (Z.C.); 2 Division of Advanced Manufacturing, Graduate School at Shenzhen, Tsinghua University, Beijing 518000, China; libh16@mails.tsinghua.edu.cn

**Keywords:** low temperature direct writing, 3D printing, LiFePO_4_, lithium-ion battery

## Abstract

LiFePO_4_ (LFP) is a promising cathode material for lithium-ion batteries. In this study, low temperature direct writing (LTDW)-based 3D printing was used to fabricate three-dimensional (3D) LFP electrodes for the first time. LFP inks were deposited into a low temperature chamber and solidified to maintain the shape and mechanical integrity of the printed features. The printed LFP electrodes were then freeze-dried to remove the solvents so that highly-porous architectures in the electrodes were obtained. LFP inks capable of freezing at low temperature was developed by adding 1,4 dioxane as a freezing agent. The rheological behavior of the prepared LFP inks was measured and appropriate compositions and ratios were selected. A LTDW machine was developed to print the electrodes. The printing parameters were optimized and the printing accuracy was characterized. Results showed that LTDW can effectively maintain the shape and mechanical integrity during the printing process. The microstructure, pore size and distribution of the printed LFP electrodes was characterized. In comparison with conventional room temperature direct ink writing process, improved pore volume and porosity can be obtained using the LTDW process. The electrochemical performance of LTDW-fabricated LFP electrodes and conventional roller-coated electrodes were conducted and compared. Results showed that the porous structure that existed in the printed electrodes can greatly improve the rate performance of LFP electrodes.

## 1. Introduction

In recent years, as 3D printing technology advances, its application has extended from mechanical components to functional devices including energy storage devices [1], biomedical devices [2,3,4], electrical/electronic devices [5,6], and so forth. These emerging applications may have great impacts on the current design methodologies of functional devices. With the aid of 3D printing, 3D architectures that were impossible to fabricate in the past have now become very simple and convenient to prepare. Device performance can be greatly enhanced based on novel and unconventional 3D-printed architectures. For energy storage devices, there have been several demonstrations of 3D-printed devices including lithium-ion batteries [7,8,9,10], super capacitors [11] and redox flow cells [12], among others. Among all these applications, 3D-printed lithium-ion batteries have drawn much attention due to its wide applications and enormous impacts on modern society [13,14,15].

In a conventional lithium-ion battery, cathode and anode plates are fabricated by roller coating of active materials on current collectors, and then parallel layer-stacking or winding with each other [16]. Therefore, conventional batteries are fundamentally 2D designs. However, 2D design has unavoidable challenges due to the trade-off between energy density and power density [17]. Three-dimensional (3D) lithium-ion battery architectures have been proposed to tackle this issue [18]. In a 3D lithium-ion battery, energy density can be improved by increasing the electrode height to accommodate more active materials while maintaining the Li-ion diffusion length constant. Therefore, the energy density can be enhanced without sacrificing power density [19,20,21].

To date, 3D lithium-ion batteries have been produced using many technologies including lithographic methods, electro-deposition, atomic layer deposition and various template synthesis methods [17]. Although significant progress has been achieved, the fabrication of 3D lithium-ion battery in a simple and low-cost way remains a challenge.

In recent years, some research groups have tried to fabricate 3D lithium-ion batteries using 3D printing due to its convenience and declining cost. J. A. Lewis’s group demonstrated the first 3D-printed 3D lithium-ion battery [10]. 3D interdigitated lithium-ion batteries composed of LiFePO_4_ (LFP) cathode and Li_4_Ti_5_O_12_ (LTO) anode were manufactured. Liangbing Hu’s group developed graphene oxide-based LFP/LTO inks to improve the electrical conductivity of LFP and LTO electrodes [7]. They also developed solid-state electrolyte inks to achieve all-component 3D-printed lithium-ion batteries. Feng Pan’s group developed a novel ink LiMn_0.21_Fe_0.79_PO_4_ (LMFP) with doping of Mn and carbon coating [8]. 3D-printed LMFP cathode was fabricated and showed impressive electrical performance such as high capacity and rate performance. Ryan R. Kohlmeyer et al. developed a universal approach to fabricate 3D-printable lithium-ion battery electrodes [9]. Well-dispersed mixture of active materials, carbon nanofibers and polymer were used to make printable electrode inks. By tuning the ratios of these components, LFP and LTO electrodes can be fabricated using direct ink writing process.

Previous research mainly focused on the development of novel inks based on the modification of active materials to improve the electrical performance of 3D-printed lithium-ion batteries. Besides inks, 3D-printing technologies also play an important role. To date, room temperature extrusion-based direct ink writing process has been used to fabricate 3D electrodes. For successful deposition of electrode ink layer upon layer, it is crucial to maintain the structural integrity and strength of printed features during the printing process. However, the electrode ink flows easily and it is difficult to maintain the shape. To solve the problem, J. A. Lewis’s group developed a graded volatility solvent system in which water evaporation induces partial solidification to ensure the shape and structural integrity of printed features [10].

In our study, low temperature direct writing (LTDW)-based 3D printing was used to fabricate 3D LFP electrodes for the first time. LFP ink was deposited into a low temperature chamber (−40 degrees Celsius) and froze as the ink was printed layer by layer. Then the frozen electrode was further freeze-dried to obtain a porous 3D electrode. Unlike the previously mentioned studies that used the evaporation of solvent to induce ink solidification, low temperature was used to freeze the electrode and maintain its shape and structural integrity in the present study. LTDW was usually used to fabricate biomaterial scaffolds with interconnected pores and graded porosity [22,23,24]. In this study, 3D-printed LFP electrode was fabricated by LTDW. LFP inks capable of freezing at low temperature were prepared and the rheological behavior were measured. The printing process was optimized and printing accuracy variation with processing parameters was obtained. Printing results were compared between LTDW and conventional room temperature extrusion-based printing process. Results showed that LTDW can significantly improve the structural integrity while serious collapse of printed features was observed with conventional method. The microstructure, pore size and distribution were characterized using -scanning electron microscope (SU-70, Hitachi, Tokyo, Japan), N_2_ adsorption isotherms (ASAP2020, Micromeritics, Norcross, GA, USA) and mercury porosimetry (AutoPore IV, Micromeritics, Norcross, GA, USA). Results showed that highly-porous 3D LFP electrodes with improved porosity can be obtained using LTDW. The improved porosity can promote the infiltration and contact of electrolyte with electrode active materials and, therefore, is beneficial to the lithium-ion diffusion and intercalation process which greatly influence the rate performance of lithium-ion batteries.

## 2. Experimental

### 2.1. Materials Preparation

To obtain a printable LiFePO_4_ ink capable of freezing at low temperature, the ink composition, ratio and preparation process need to be carefully designed. The ink composition can be divided into three parts: active materials like LiFePO_4_; additives including binders and conductive agents, and; solvents that dissolve polymer binders to make a polymer solution. Powders including active materials and conductive agents were evenly dispersed into the binder polymer solution to prepare the ink. In the composite materials, the only freezable agent is the solvent. Usually, for conventional LiFePO_4_ slurry, the solvent is NMP (*N*-methyl-2-pyrrolidone) and the binder is PVDF (poly(vinylidene fluoride)). In LTDW, freezing rate plays an important part in the freezing of the ink during the printing process. To achieve a high freezing rate, a large undercooling degree is required but is very difficult to execute for NMP with a freezing point of −24 degrees Celsius. Therefore, ink composition and ratios used in conventional lithium-ion batteries are not applicable in LTDW process and new materials need to be developed.

In our study, 1,4 dioxane was added into the solvent system and functioned as the freezing agent due to its high freezing point (11.8 degrees Celsius) and low specific heat capacity. 1,4 dioxane and deionized water were mixed to obtain a mixed solution. CMC (carboxy methyl cellulose) was used as the binder to be dissolved in the mixed solution. Then, LiFePO_4_ powder was added and stirred by a vacuum mixer to obtain the prepared ink. To avoid possible clogging due to agglomeration, the ink was filtered by a mesh screen to remove large clusters.

### 2.2. LTDW Process

**Figure 1:**
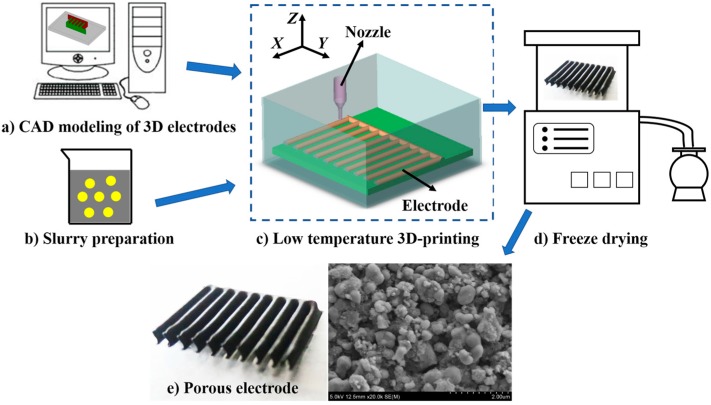
Fabrication process of LiFePO_4_ (LFP) electrode by low temperature direct writing (LTDW). (**a**) CAD modeling of 3D electrodes, (**b**) slurry preparation, (**c**) low temperature direct writing process, (**d**) freeze-drying process, (**e**) porous electrodes.

The schematic of the LTDW process is shown in Figure 1. The first step is CAD modeling of 3D electrodes using CAD software (Solidworks, Dassault Systemes, Paris, France). Then the CAD model was digitally sliced into layers to obtain the slice file. Ink was loaded into a syringe and extruded through a micro-nozzle and deposited layer by layer according to the CNC (computer numerical control) paths of the slice file. Then the printed electrode was transferred to a freeze-drying machine to remove the solvents and a porous electrode was obtained.

**Figure 2:**
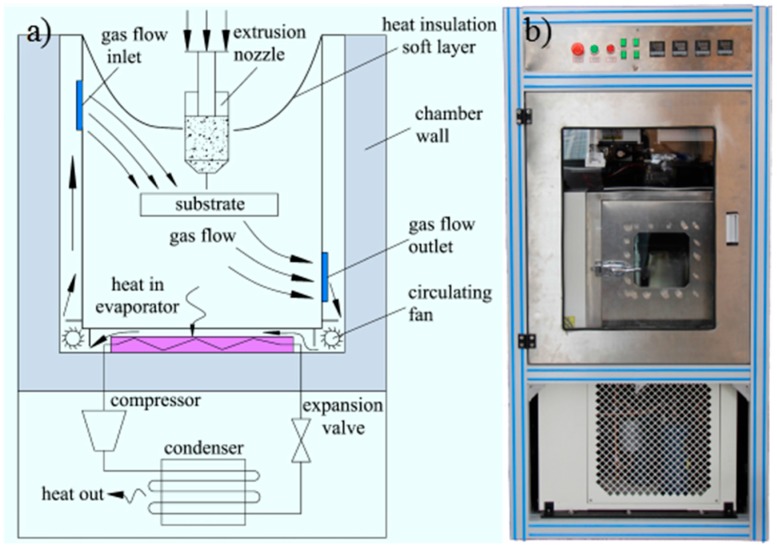
Schematic and image of the LTDW machine. (**a**) Schematic of the LTDW machine, (**b**) the LTDW machine.

Toward this goal, a LTDW machine (Shenzhen University, Shenzhen, Guangdong, China) (shown in Figure 2) composed of a low temperature chamber, an extrusion nozzle, a *XYZ* motion stage, a CNC control system and GUI software was developed. The printing head was equipped with a heating unit to avoid the solidification of LFP inks. The LFP ink loaded in the syringe was extruded by a step motor-driven extrusion rod and deposited into the low temperature chamber. With the scanning of printing head in the *XY* direction, printed LFP materials lines are deposited layer by layer to form 3D porous electrodes.

### 2.3. Characterization

Rheological behavior of the prepared LFP inks was measured using rotational rheometer (AR1000, TA Instruments, New Castle, DE, USA). Appropriate compositions and ratios of LFP inks were selected according to the rheological results. The accuracy of the printed electrodes was characterized using an advanced optical 3D microscope (VHX-5000, Keygence, Osaka, Japan). The printing parameters influencing the printing accuracy were examined and optimal printing parameters were obtained. Detailed microstructures of the printed electrodes was characterized using a scanning electron microscope (SU-70, Hitachi, Tokyo, Japan). Nitrogen adsorption isotherms were measured on ASAP 2020(Micromeritics, Norcross, GA, USA). The specific surface area was computed using the multipoint Brunauer-Emmett-Teller (BET) method. Pore size and distribution was computed using the Barrett-Joyner-Halenda (BJH) method. Since nitrogen adsorption method was only capable of characterizing pores in the diameter rang of 2 nm~200 nm. Mercury porosimetry was performed to measure the pore size, distribution and porosity on mercury intrusion machine (AutoPore IV, Micromeritics, Norcross, GA, USA).

To verify the impact of the porous structure on the rate performance of LiFePO_4_ electrodes, the rate performance of LiFePO_4_ electrodes fabricated by LTDW process and conventional roller coating method was compared. The electrodes were assembled into coin-type half-cells using Li-metal as the counter electrode. The charge/discharge performance was conducted at cutoff voltage between 2.5 and 4.2 V. The current rate was 0.1, 0.2, 0.5, 1, 5, and 10 C. Cycle performance was also studied at 0.5 C for 100 cycles.

## 3. Results

### 3.1. Rheological Properties of LFP Slurries

**Figure 3:**
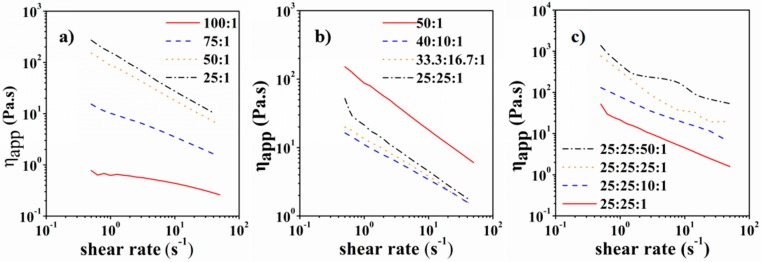
Rheological properties of LFP inks: (**a**) carboxy methyl cellulose (CMC) water solution; (**b**) CMC-water-1,4 dioxane solution; (**c**) LFP-CMC-water-1,4 dioxane ink.

CMC is a commonly used binder for lithium-ion batteries. In addition to binding the LFP particles, CMC can also be used to adjust the viscosity of the LFP ink that is very important to its printability. To investigate the influence of CMC content on the apparent viscosity of LFP ink, CMC aqueous solutions with the loading of 1 wt %, 1.33 wt %, 2 wt %, and 4 wt % were prepared. Results showed that with the increase of CMC content, the viscosity increased dramatically (shown in Figure 3). When the CMC content reached 2%, the apparent viscosity varied between 10 Pa·s and 100 Pa·s (shear rate 1~50 S^−1^). The adding of 1,4 dioxane to the solvent system had an important impact on the solution properties. The apparent viscosity of CMC-water-1,4 dioxane solution was greatly reduced (shown in Figure 3). The apparent viscosity increased with the increase of 1,4 dioxane proportion in the solvent system. When the 1,4 dioxane proportion exceeded 50%, white flocs were observed as CMC could not completely dissolve in the solvent. As a result, 50% proportion was selected. Then LFP particles were mixed into the CMC-water-1,4 dioxane solution to obtain the LFP ink. The apparent viscosity increased remarkably with the increase of LFP amount. When the amount of LFP powder reached 25 g/50 mL, the apparent viscosity of LFP ink varied between 50 Pa·s and 500 Pa·s (shear rate 1~50 S^−1^). If the amount of LFP exceeded 25 g/50 mL, the ink tended to dry very quickly and the dried particles greatly affected the printing fluency and continuity. After careful evaluation, LFP ink with 25 g LFP powder dispersed in 50 mL CMC solution (25 mL water and 25 mL 1,4 dioxane) was selected for the LTDW process. In our experiments, the printable maximum and minimum mass loading of active materials in the LFP slurry was 30 g/50 mL and 15 g/50 mL, respectively.

### 3.2. Printing Process Optimization and Accuracy Characterization

**Figure 4:**
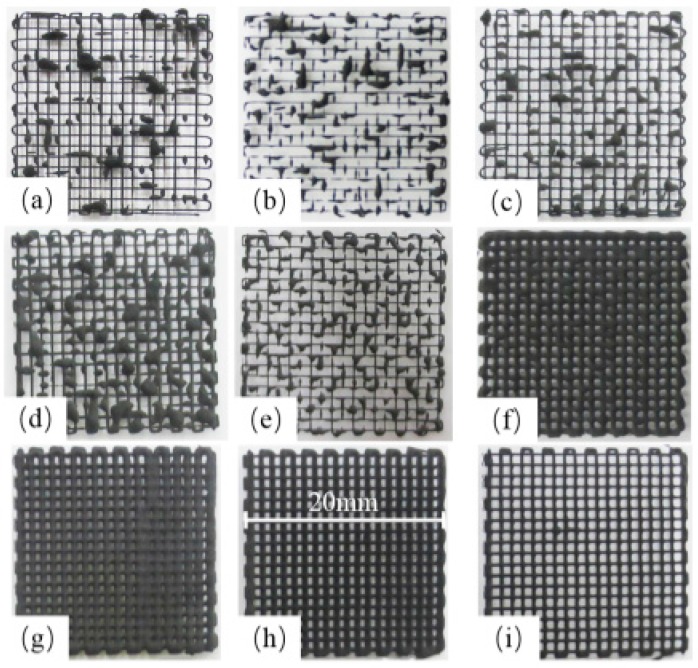
Images of printed LFP grid patterns using different printing parameters. (**a**) *v_j_* = 0.003 mm/s, *v_xy_* = 6 mm/s, *h_s_* = 0.2 mm; (**b**) *v_j_* = 0.003 mm/s, *v_xy_* = 4 mm/s, *h_s_* = 0.15 mm; **c**) *v_j_* = 0.009 mm/s, *v_xy_* = 6 mm/s, *h_s_* = 0.15 mm; (**d**) *v_j_* = 0.006 mm/s, *v_xy_* = 4 mm/s, *h_s_* = 0.2 mm; (**e**) *v_j_* = 0.006 mm/s, *v_xy_* = 6 mm/s, *h_s_* = 0.1 mm; (**f**) *v_j_* = 0.009 mm/s, *v_xy_* = 4 mm/s, *h_s_* = 0.1 mm; (**g**) *v_j_* = 0.009 mm/s, *v_xy_* = 2 mm/s, *h_s_* = 0.2 mm; (**h**) *v_j_* = 0.006 mm/s, *v_xy_* = 2 mm/s, *h_s_* = 0.15 mm; (**i**) *v_j_* = 0.003 mm/s, *v_xy_* = 2 mm/s, *h_s_* = 0.1 mm.

For the LTDW process, main printing parameters include: extrusion speed *v_j_*, scanning speed *v_xy_* and layer thickness *h_s_*. To investigate the influence of printing parameters on the printing results, an orthogonal test scheme L_9_(3^3^) was designed (shown in Table 1). Printing tests were carried out using a grid pattern with the dimensions of 20 mm × 20 mm × 2 mm with line spacing of 1 mm. The printing results are shown in Figure 4.

It can be seen that the printing parameters have important impacts on the fabrication results. From the printed patterns shown in Figure 4a–e, disconnection and discontinuity of the printed lines can be observed as a result of non-uniform materials supply caused by inappropriate combination of printing parameters. In Figure 4f–i, by contrast, clear and continuous printed patterns can be seen. However, the printed line width varied with different combinations of printing parameters. Figure 4i exhibits the best printing accuracy with the finest line width.

**Figure 5:**
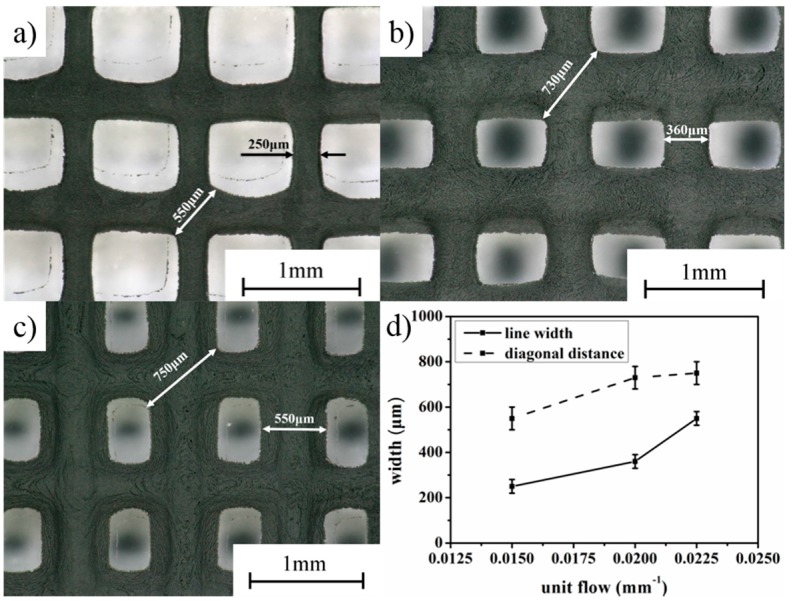
Comparison on the printing accuracy of LTDW printed grid patterns using different parameters: (**a**) *v_j_* = 0.003 mm/s, *v_xy_* = 2 mm/s, *h_s_* = 0.1 mm; (**b**) *v_j_* = 0.006 mm/s, *v_xy_* = 2 mm/s, *h_s_* = 0.15 mm; (**c**) *v_j_* = 0.009 mm/s, *v_xy_* = 4 mm/s, *h_s_* = 0.1 mm; (**d**) variation of line width and diagonal distance with unit flow equivalent number.

To characterize the printing accuracy, the values of line width and diagonal distance of the intersection point are shown in Figure 5. Three groups of parameters displayed in bold in Table 1 with good printing quality are compared. A unit flow equivalent number was defined as: *v_j_*/(*v_xy_* × *h_s_*). The number denotes that unit flow is proportional to the extrusion speed and inversely proportional to the scanning speed and layer thickness. The variation of line width and diagonal distance with equivalent number is shown in Figure 5d. It can be seen that both line width and diagonal distance increased with the increase of the equivalent number. The best line width of 250 μm using parameters *v_j_* = 0.003 mm/s, *v_xy_* = 2 mm/s, *h_s_* = 0.1 mm can be obtained.

**Figure 6:**
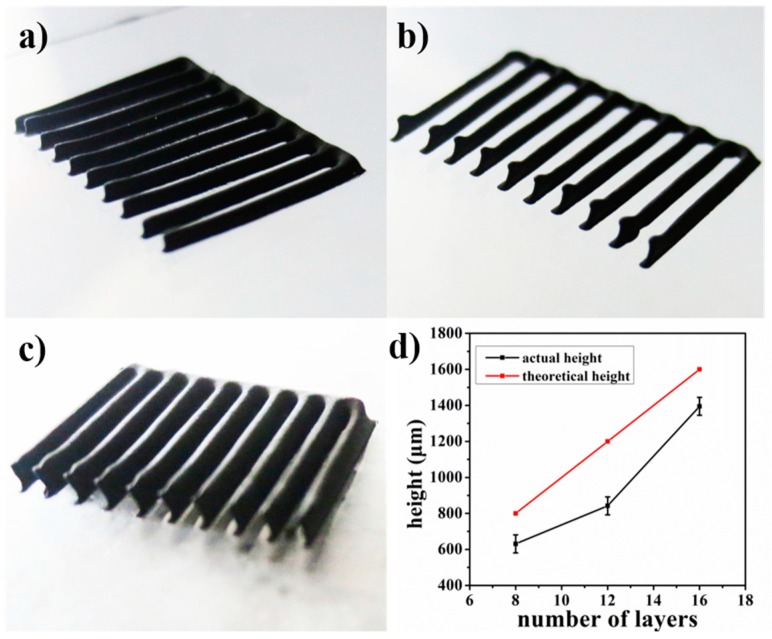
Printed comb-shaped LFP electrode: (**a**) 8 layers; (**b**) 12 layers; (**c**) 16 layers; (**d**) electrode height variation with number of layers.

To further characterize the printing accuracy on the height direction, a comb-shaped LFP electrode was printed and shown in Figure 6. The LFP electrodes were printed with 8 layers, 12 layers and 16 layers, respectively. Then the height of LFP electrodes was measured, and the variation of electrode height with the number of layers is shown in Figure 6d. It can be seen that the actual height of printed electrodes was about 85% of the theoretical values, indicating the existence of possible material spreading. We guess there are some factors in the whole process that may lead to the spreading of material, including the exposure of materials to the atmosphere and the freeze-drying process. Although the spreading of materials may be difficult to eliminate, the height reduction did not influence the printed shape and the mechanical integrity can be well maintained.

**Figure 7:**
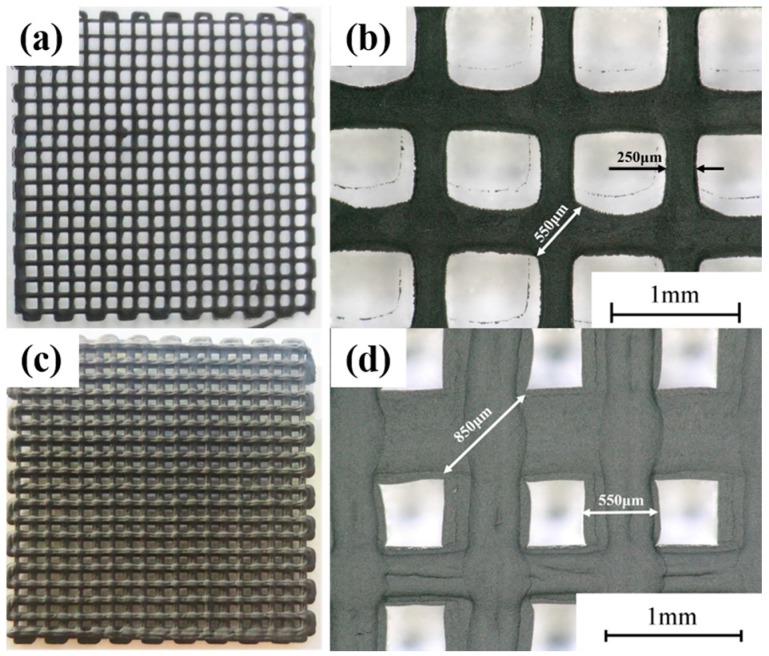
Comparison of LTDW printed patterns and room temperature printed patterns: (**a**) LTDW printed patterns; (**b**) the printing accuracy of LTDW printed patterns; (**c**) room temperature printed patterns; (**d**) the printing accuracy of room temperature printed patterns.

To further verify the effectiveness of LTDW in maintaining the shape of printed LFP electrodes, comparison was made between the patterns printed at low temperature and at room temperature respectively using exactly the same printing parameters. The printed results are shown in Figure 7. It can be seen that serious spreading of materials can be observed for patterns printed at room temperature. Both the line width and diagonal distance were much larger than LTDW printed ones. The comparison between the two printed patterns indicates that LTDW can effectively improve the capacity for maintain the shape and mechanical integrity in comparison with conventional room temperature methods.

### 3.3. Microstructure Characterization

**Figure 8:**
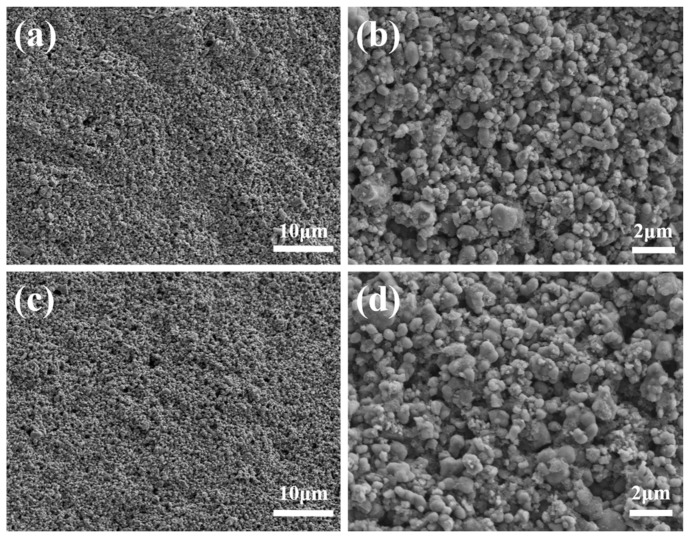
SEM images of the surface of printed LFP electrodes: (**a**,**b**) room temperature printed LFP electrodes; (**c**,**d**) LTDW printed LFP electrodes.

**Figure 9:**
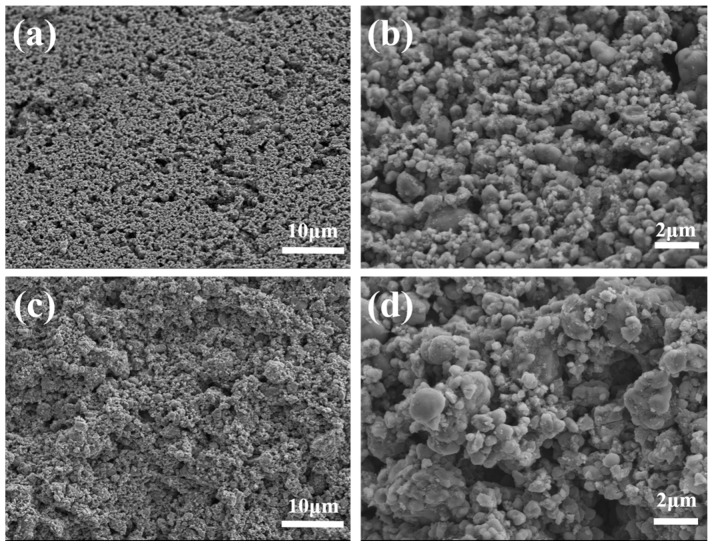
SEM images of the cross section of printed LFP electrodes: (**a**,**b**) room temperature printed LFP electrodes; (**c**,**d**) LTDW printed LFP electrodes.

To characterize the microstructure of the 3D-printed LFP electrodes, SEM images were taken on the surface and cross section of both electrodes, as shown in Figure 8 and Figure 9, respectively. Porous features can be observed both on the surface and the cross section. The LFP particles were bonded by CMC binders. The diameter of LFP particles ranges from 1μm to 10μm. The pores and voids between LFP particles that provide space for electrolyte can be observed and have very important impacts on the electrochemical performance.

### 3.4. Characterization of Pore Volume, Size and Distribution

**Figure 10:**
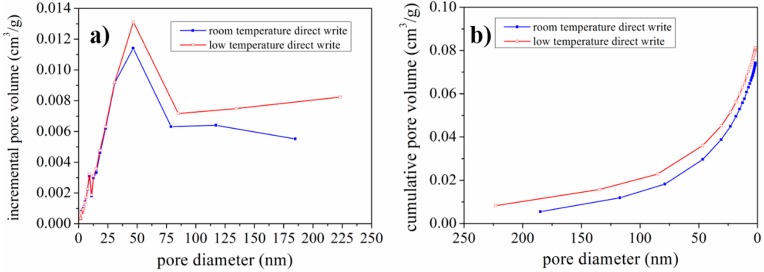
Pore size and distribution measured by nitrogen adsorption: (**a**) incremental pore volume variation with pore diameter; (**b**) cumulative pore volume variation with pore diameter.

To further characterize the pore size and distribution, nitrogen adsorption isotherms were measured. The incremental pore volume variation with pore diameter and cumulative pore volume varied with pore diameter are shown in Figure 10. It can be seen that the incremental pore volume for pores of diameter less than 30 nm is approximately the same for LTDW and room temperature printed electrodes. For pores of diameter over 50 nm, LTDW printed electrodes had larger pore volume than room temperature printed electrodes. From the cumulative pore volume curves, it can be seen that the total pore volume of LTDW printed electrodes was larger than room temperature printed electrodes. The results indicate that LTDW can be used to print LFP electrodes with improved pore volume. Besides pore volume, the BET (Brunauer-Emmett-Teller) specific surface area was also obtained. The specific surface area value was 28.03 m^2^/g for room temperature printed electrodes and 28.13 m^2^/g for LTDW printed electrodes, which are very close. Since the specific surface area was mainly determined by the diameter of LFP particles, the fabrication process had little impact.

**Figure 11:**
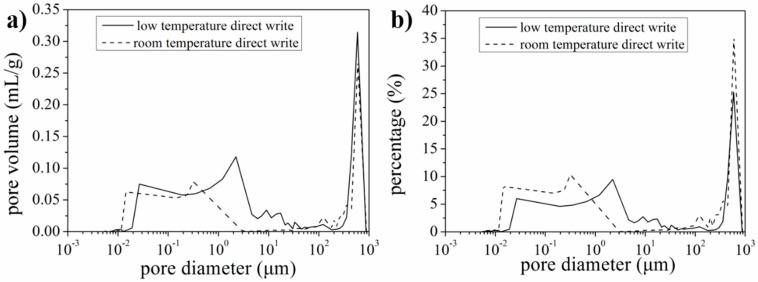
Pore size and distribution measured by mercury porosimetry: (**a**) pore volume variation with pore diameter; (**b**) percentage distribution of pores variation with pore diameter.

The SEM images shown in Figure 8 and Figure 9 revealed that a large number of pores with diameter over 1 μm existed in the LFP electrodes. Since the nitrogen adsorption isotherms were only capable to characterize the pores in the diameter range of 2~200 nm. Therefore, in order to better characterize the pore size and distribution, mercury porosimetry capable of measuring pores in the diameter range from tens of nanometers to hundreds of micrometers was performed. Pore volume variation with pore diameter is shown in Figure 11a and percentage distribution of pores variation with pore diameter is shown in Figure 11b. It can be seen that the volume of pores of diameter below 200 nm for LTDW printed electrodes is slightly larger than room temperature printed electrodes. The volume of pores of diameter over 50 μm is approximately identical for electrodes fabricated using both processes. In the diameter range of 200~50 μm, the pore volume of LTDW printed electrodes is much larger than room temperature printed ones. The porosity of printed electrodes was also obtained by the mercury intrusion process. Results showed that the porosity of the LTDW printed electrodes was 71.8%, while the porosity was 61.4% for room temperature printed ones.

### 3.5. Electrochemical Performance

**Figure 12:**
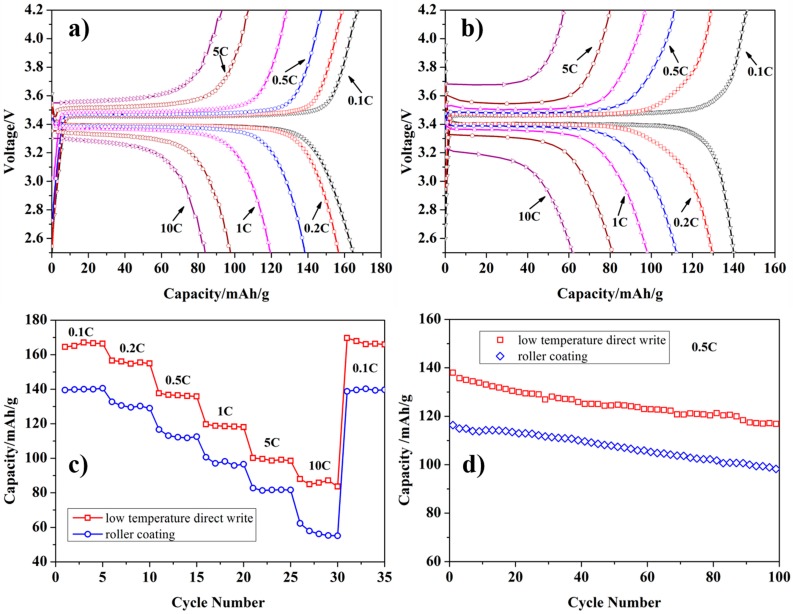
Electrochemical performance of LTDW-fabricated LiFePO_4_ electrodes and conventional roller-coated electrodes: (**a**) charge/discharge curves of LTDW-fabricated electrodes; (**b**) charge/discharge curves of roller-coated electrodes; (**c**) rate performance; (**d**) cycle performance.

To verify the effectiveness of the porous structure in improving the rate performance of LiFePO_4_ electrodes, the electrochemical performance of the electrodes fabricated by LTDW process and the conventional roller coating process was compared. To make the results comparable, the mass of the active materials coated on the aluminum foil for both of the two types of electrodes was adjusted to around 4.0 mg. Figure 12 shows the charge/discharge curves, rate performance and cycle performance of the two types of electrodes.

It can be seen that the discharge capacities at various current rates (0.1~10 C) of the LTDW-fabricated electrodes have been greatly enhanced in comparison with conventional ones fabricated by roller coating. When the rate is 10 C, the discharge capacity is 61 mAhg^−1^ for conventional electrodes while 82 mAhg^−1^ for LTDW-fabricated electrodes. Cycle performance was measured at 0.5 °C for 100 cycles. The capacity retention is around 85% for both types of electrodes after 100 cycles. Electrochemical results showed that the porous structure achieved using the LTDW process can improve the rate performance of LiFePO_4_ electrodes.

In comparison with other studies that tried to improve the rate performance of LiFePO_4_ by materials modification and novel synthesis approach which can improve the discharge capacity to over 100 mAhg^−1^ (10 C) [25,26], the rate performance presented in this study is not charming. There are two reasons limiting the improvement of rate performance. One is that the average particle size of commercial LiFePO_4_ powder used in this study was ~2 μm and larger diameter of particle would limit the transport of Li-ions in solid active materials. Another important factor is the binder CMC used in our study. Usually, PVDF is the most commonly used binder for LiFePO_4_. We speculate that the combination of active materials and binders led to the relatively poor rate performance obtained in this study. However, the LTDW process which can produce highly porous electrodes may be used to improve the rate performance of lithium-ion batteries.

## 4. Discussion

From the quality of the printed results and the characterization of the printed electrodes, it can be concluded that LTDW is an effective process for fabricating 3D LFP electrodes. The idea of freezing LFP ink during the printing process to maintain the mechanical integrity and the shape of the printed features is viable and practical. In comparison with previously reported printing strategy by rapid solvent partial evaporation enabled by a specially developed graded solvent system, the height reduction (around 15%) in this study is slightly larger, which is considered to be mainly due to the exposure of frozen electrode to the atmosphere and spread of materials during the freeze-drying process. However, the printed results have demonstrated the capacity of maintaining the shape and mechanical integrity. From the characterization of pore size and distribution using nitrogen adsorption isotherms and mercury intrusion process, it can be seen that LTDW can be used to fabricate 3D LFP electrodes with improved porosity and pore volume. The enhanced pore volume and porosity can provide more space for electrolyte accommodation and infiltration, which is beneficial to the rate performance of lithium-ion batteries. Electrochemical performance demonstrated the effectiveness of porous structure on improving the discharge capacity at high current rates. Gen Inoue et al. [27] conducted an numerical and experimental evaluation of the relationship between porous electrode structure and effective conductivity of ions in lithium-ion batteries. It showed that in a macroscopic porous electrode, the effective ionic conductivity is proportional to the porosity of electrodes and the increase of porosity can greatly enhance the transport of Li-ions. This coincides with the results presented in this study.

## 5. Conclusions

A LTDW-based 3D printing process was used to fabricate 3D LFP electrodes for the first time. A LTDW machine was developed and LFP inks capable of freezing at low temperature were developed. 1,4 dioxane was added as a freezing agent to meet the LTDW process requirements. The rheological behavior of the LFP ink was measured and appropriate viscosity range was obtained. Printing parameters were optimized by an orthogonal experiment and the optimal printing parameters were obtained. The printing accuracy was characterized and the printed line width and diagonal distance variation with unit flow equivalent number was obtained. Printed results showed that LTDW process can effectively maintain the shape and mechanical integrity of the printed structures, while room temperature printed electrodes cannot. The comparison demonstrates the feasibility and capacity of LTDW in fabricating 3D LFP electrodes. SEM characterization on the microstructures of printed electrodes using LTDW process and room temperature printing process was performed. Results showed that porous electrodes can be fabricated using both processes. Nitrogen adsorption isotherms and mercury porosimetry were used to characterize the pore size and distribution of the printed 3D LFP electrodes. Results showed that improved pore volume and porosity were obtained by the LTDW process. The process can be used to fabricate highly porous 3D LFP electrode and the improved electrode porosity can enhance the rate performance, which was verified by the electrochemical results.

## Figures and Tables

**Table 1:** Printing parameters.

No.	Extrusion Speed *v_j_*(mm/s)	Scanning Speed *v_xy_*(mm/s)	Layer Thickness *h_s_*(mm)	Printed Structures (Figure 4)
1	0.003	2	0.1	(i)
2	0.003	4	0.15	(b)
3	0.003	6	0.2	(a)
4	0.006	2	0.15	(h)
5	0.006	4	0.2	(d)
6	0.006	6	0.1	(e)
7	0.009	2	0.2	(g)
8	0.009	4	0.1	(f)
9	0.009	6	0.15	(c)
